# Efficacy and safety of Dexrazoxane (DRZ) in sarcoma patients receiving high cumulative doses of anthracycline therapy – a retrospective study including 32 patients

**DOI:** 10.1186/s12885-016-2654-x

**Published:** 2016-08-09

**Authors:** Markus K. Schuler, Sebastian Gerdes, Antje West, Stephan Richter, Christoph Busemann, Leopold Hentschel, Felicitas Lenz, Hans-Georg Kopp, Gerhard Ehninger, Peter Reichardt, Daniel Pink

**Affiliations:** 1 Department of Internal Medicine I, University Hospital Carl Gustav Carus, Technical University Dresden, Fetscherstraße 74, 01307 Dresden, Germany; 2 Department of Internal Medicine II, HELIOS Clinic Emil von Behring, Walterhöferstr. 11, 14165 Berlin, Germany; 3 Institute of Medical Informatics and Biometry, University Hospital Carl Gustav Carus, Technical University Dresden, Fetscherstraße 74, 01307 Dresden, Germany; 4 Department of Hematology and Oncology, HELIOS Clinic Bad Saarow, Pieskower Straße 33, 15526 Bad Saarow, Germany; 5 Department of Internal Medicine C, University Hospital Greifswald, Sauerbruchstraße, 17475 Greifswald, Germany; 6 University Cancer Center, University Hospital Carl Gustav Carus, Technical University Dresden, Fetscherstraße 74, 01307 Dresden, Germany; 7 Department of Internal Medicine II, University Hospital Tübingen, Eberhard Karls University Tübingen, Geissweg 3, 72076 Tübingen, Germany; 8 Department of Interdisciplinary Oncology, HELIOS Clinic Berlin-Buch, Berlin, Germany

**Keywords:** Soft tissue sarcoma, Advanced disease, Palliative care, Anthracyclines, Dexrazoxane, Cardiotoxicity

## Abstract

**Background:**

Anthracyclines, as the most effective therapy, are the cornerstone of advanced stage sarcoma treatment. However, anthracyclines can also contribute to myocardial dysfunction and congestive heart failure, ultimately limiting the therapeutic potential of the drug. Coadministration of Dexrazoxane has been shown to effectively reduce cardiotoxicity, however primarily in patients suffering in diseases other than sarcoma.

**Methods:**

The aim of this retrospective analysis was to evaluate safety and efficacy of chemotherapy with high cumulative doses of anthracyclines in combination with Dexrazoxane. The medical charts of 32 patients treated in four institutions were analyzed. Reasons for coadministration were rechallenge, reaching the cumulative anthracycline dose and preexisting heart failure.

**Results:**

The median age was 54 years [18–68 years]. The median cumulative anthracycline dose before adding DRZ was 450 mg/m^2^ and after administration of last anthracycline containing therapy 750 mg/m^2^. Either during treatment or follow up, 2/27 patients (7 %) without preexisting major cardiac findings developed anthracycline-induced cardiotoxicity. The median overall survival (OS) from start of the first anthracycline containing chemotherapy was 46 months and 17 months from the initial coadministration of DRZ. At rechallenge, the median progression free survival (PFS) with DRZ was 7 months. In continuous therapy, the median PFS was 13 months from beginning of chemotherapy and 9 months from the addition of DRZ.

**Conclusion:**

Chemotherapy with high cumulative doses of anthracyclines in addition with DRZ demonstrated a remarkable OS in these advanced disease patients. Cardiac side-effects due to high cumulative doses of anthracyclines requiring discontinuation of anthracycline treatment were rare. A PFS of 9 months from the beginning of the coadministration of DRZ indicates that continuing anthracycline therapy beyond established cumulative doses is a promising therapeutic option.

## Background

Cardiotoxicity is a major concern as a late side effect of cancer treatment with anthracyclines [1]. Among several cancer therapies responsible for cardiotoxicity, anthracyclines predominantly cause myocardial dysfunction and congestive heart failure (CHF). Anthracyclines’ side effects present a great concern for sarcoma patients, since anthracyclines remain to be the most effective therapy in advanced stage sarcoma treatment [2].

The understanding of the molecular pathogenesis of anthracycline-induced cardiotoxicity (AICM) remains partly controversial. Cardiac tissue contains a weak antioxidant activity and oxygen-derived free radicals most likely contribute to the cardiotoxic effect [3, 4]. Acute cardiotoxicity is not dose dependent and usually has an early onset. Clinical manifestations include sinus tachycardia and supraventricular or ventricular arrhythmia, which are mostly self-limiting and present no clinical harm. More concerning are the late-onset cardiotoxic effects, most often exerted by former use of anthracycline therapy. Originally, AICM has been defined by the deterioration of left ventricular dysfunction, detectable by echocardiogram [5]. Today, cardiac dysfunction is defined by left ventricular dysfunction or CHF [6]. The probability of cardiomyopathy induced by doxorubicin (DOX), the most widely used anthracycline, is clearly related to the cumulative dose. Moreover, age and preexisting cardiac injuries also are known as independent risk factors for doxorubicin-related CHF [7]. The estimated risk in adult patients increases up to 26 % at a cumulative doxorubicin dose of 550 mg/m^2^, which is in contrast to an estimated 7 % in an adolescent cancer patient population [8].

There have been many different attempts to minimize the cardiotoxic effects caused by anthracyclines. Besides developing new derivates and encapsulated formulations, the most promising results have been achieved by prolonged or continuous anthracycline infusions [9–11]. Furthermore, cardioprotective efficacy has been demonstrated for the iron-chelator Dexrazoxane (DRZ), which reduces free oxygen radicals [12]. The addition of DRZ to the anthracycline-based chemotherapy leads to a significantly smaller decrease in left ventricular ejection fraction (LVEF) and a lower incidence of CHF in a randomized controlled trial in children with acute lymphoblastic leukemia. Simultaneously, children with ALL experienced no differences in response rate, PFS and OS [13].

Of note, cardiac biomarkers like troponin I and BNP have been proven as valuable and early detectors of cardiotoxicity. The immediate use of cardioprotective medications might have the chance to minimize or partly reverse the damage that has been caused [14]. Recently, a randomized controlled trial with a combination of an Angiotensin-converting enzyme inhibitor (enalapril) and a beta-blocker (carvedilol) demonstrates that this combination might be effective in preventing anthracycline induced cardiomyopathy [15].

For many years, the iron chelator DRZ has been investigated in preclinical models [16] and used as a cardioprotective agent together with anthracyclines, allowing cumulative doses to be doubled [17]. Yet, in some countries, the use of DRZ has been restricted, because of the suspected reduced anticancer activity [18], and a suspected higher incidence of secondary malignancies [19]. However, several large randomized trials in hematologic malignancies (acute lymphoblastic leukemia, Hodgkin’s disease, myelodysplastic syndrome) did not confirm these hypotheses [20, 21].

There have been studies and recommendations for the use of DRZ mainly in breast cancer (BC), acute lymphatic leukemia and Hodgkin’s disease [11, 13]. A 2008 clinical practice guideline published by the American Society of Clinical Oncology (ASCO) recommends considering the use of DRZ in doxorubicin-containing regimens in patients with BC or other malignancies, who have received more than 300 mg/m^2^ doxorubicin [22]. But, so far there is only very limited information about the use of DRZ as a cardioprotective agent in soft tissue sarcoma patients, especially in adult patients. In a small randomized study with 38 pediatric sarcoma patients, Wexler et al. found that DRZ significantly reduced the risk of subclinical cardiotoxicity (22 versus 67 %) and reduced the decline in LVEF without negatively affecting rates of response [23]. Lopez and colleagues performed a randomized study in patients with breast cancer (*n* = 95) and soft tissue sarcoma (*n* = 34) [24]. Patients were randomized to receive epirubicin, another widely used anthracycline, with or without concomitantly DRZ. Results indicate that patients in the control arm experienced a significantly greater decrease in LVEF from baseline. Non-cardiac toxicity and antitumor efficacy for the two treatment groups was nearly the same. Similar results were obtained by other studies and case series [25–29].

For many patients with unresectable or metastatic sarcomas, anthracycline containing chemotherapy is one of the most effective therapeutic options. Often, the use of anthracyclines in these patients will be discontinued not due to progressive disease. In most cases, therapy is interrupted to prevent cumulative side effects, especially because of concerns regarding cumulative cardiotoxicity. On the other hand, only limited other therapeutic options are available for these patients. Due to the rarity of soft tissue sarcomas, no published data from randomized prospective trials verify the benefit of third- or forth-line therapy. However, in many experienced sarcoma centers patients with advanced disease, good performance status and no relevant comorbidities are offered more than one or two lines of therapy. Therefore, selected patients with primary responsive disease might also benefit from anthracycline rechallenge where no other effective therapeutic options are available. Thus, it seems highly interesting whether the cardioprotective effect of DRZ can be demonstrated in sarcoma patients as well. The aim of this study was to investigate the possibility of treating sarcoma patients with high cumulative anthracycline doses without clinically relevant cardiotoxicity when patients receive anthracyclines in combination with DRZ. This retrospective analysis seeks to provide more evidence in terms of the safety, efficacy and effectiveness for the use of anthracyclines in combination with DRZ in sarcoma patients.

## Methods

Clinical records of all patients with soft tissue sarcoma and metastatic or advanced disease treated with DOX and DRZ from 2000 until 2012 at the four different institutions (HELIOS Klinikum Berlin-Buch, HELIOS Klinikum Bad Saarow, Universitätsmedizin Greifswald, and Universitätsklinikum Dresden) were retrospectively reviewed. Patients treated with DRZ after anthracycline extravasation to prevent tissue necrosis were excluded from this analysis. In addition to tumor and demographic variables, all safety and efficacy data were collected. Follow-up was continued until death or up until last patient contact. Due to the retrospective nature of the analysis, no analysis-specific informed consent could be obtained. General consent for analysis and publication was obtained by the contract governing medical treatment.

DRZ was given intravenously 30 min before the start of doxorubicin over a period of 15 min in tenfold concentration compared with DOX dosage. All doxorubicin regimens were combination regimens with dacarbazine (DOX 60–75 mg/m^2^ day 1 and dacarbazine 400 mg/m^2^ day 1–3).

For response evaluation “Response Evaluation Criteria in Solid Tumors” (RECIST 1.1) were applied [30]. Criteria measuring AICM were defined as meeting one of the following clinical or functional criteria: new onset of symptoms of heart failure or new hypokinesia, drop in left ventricular ejection fraction below 50 %, or decrease of more than 10 % from basal value measured by transthoracal echocardiography (TTE). New diastolic relaxation disturbances by itself were not considered AICM.

## Results

### Patient characteristics

Medical records of 32 patients (15 male, 17 female) who received DRZ were evaluated in this series. The median age at first presentation was 54 years [18 – 68 years]. Ten patients had extremity tumors and 21 patients had tumors of the trunk. In one patient, the primary site was unknown. The most common subtypes included leiomyosarcoma (*n* = 12) and liposarcoma (*n* = 7). Common metastatic sites were lung (*n* = 22), liver (*n* = 13) and bone (*n* = 11). The median age at the beginning of therapy with DRZ was 57.5 years [22–69 years].

Reasons for using DRZ were prevalent cardiac injury (CIN) as defined by the personal assessment of the treating physician, rechallenge (RC) with anthracyclines, or reaching the anthracycline cumulative dose (CD) with an increased risk of AICM.

**Fig. 1:**
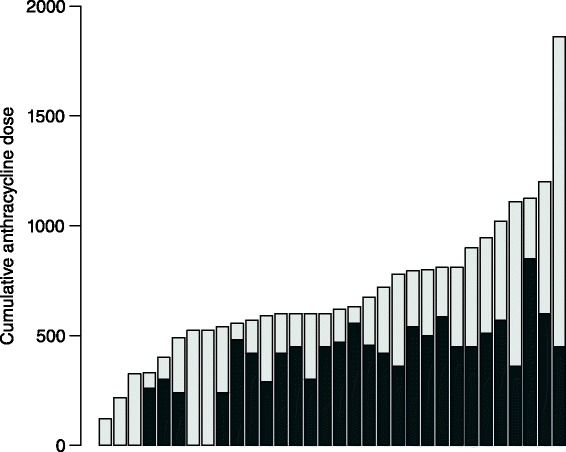
Cumulative anthracycline dosage. Each patient is represented by a vertical bar. The patients are sorted by total cumulative dosage. In the light gray part, DRZ was coadministered

Five patients received DRZ prophylactic upfront during anthracycline containing therapy. By judgment of the treating physician, the patients were not eligible for a conventional regimen due to one the following reasons: CHF, severe arrhythmia, or coronary heart disease. In those five patients, the median cumulative anthracycline dose delivered was 325 mg/m^2^ [120–525 mg/m^2^] (Fig. 1). The median PFS was 13 months [0–24 months]. After a median follow-up of 22 months, three patients were still alive with no evidence of congestive heart failure. All patients evaluable during follow-up showed no decrease in LVEF.

**Fig. 2:**
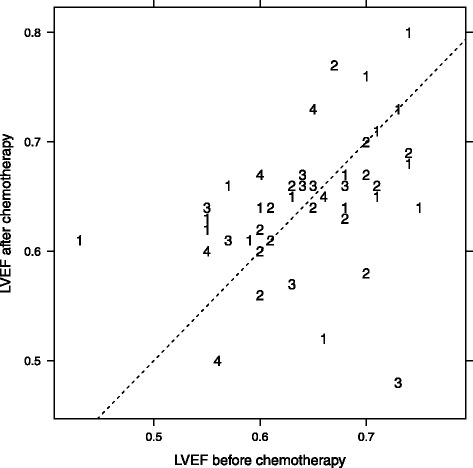
Left ventricular ejection fraction before and after chemotherapy for all chemotherapies containing anthracyclines. Note that a single patient can have received more than one chemotherapy containing anthracyclines. The diagonal line has slope 1, hence points above the line indicate a higher LVEF after chemotherapy, and vice versa. The number indicates the line of treatment of the chemotherapy that has been analyzed

Twenty-seven patients received DRZ in prophylactic intention (RC and CD), with 2 patients meeting the criteria of both groups. In the subgroup analysis they were therefore evaluated separately. None of them had a preexisting cardiomyopathy. The median LVEF was 65.5 % [54–77 %]. Overall, the main prevalent cardiac findings were diastolic relaxation disturbances (11 patients), mild left ventricular hypertrophy (7 patients) and grade I-II valvular insufficiency (8 patients). The median cumulative DOX dose before adding DRZ was 450 mg/m^2^ [range: 240–850 mg/m^2^]; after administration of last DOX containing therapy in combination with DRZ, the median cumulative DOX dose was 750 mg/m^2^ [range: 330–1860 mg/m^2^] (Fig. 1). During treatment and follow-up, no decrease in LVEF was detected in 12 patients. Thirteen patients showed a slight decrease of LVEF (median of 5 % [1–27 %]) and 2 patients were not evaluable due to missing echocardiography (Fig. 2).

In this group of 27 patients, the median PFS from starting the DRZ containing chemotherapy was 9 months [0–25 months] and the median OS from starting DRZ comedication was 17 months [1–60 months].

During treatment or follow up, 2/27 patients (7 %) without preexisting major cardiac findings developed AICM. One patient had a significant decrease in LVEF while receiving DRZ. Before DRZ coadministration was initiated, the pretreatment cumulative DOX dose equivalent was 300 mg/m^2^. After two cycles of DOX/dacarbazine, echocardiography revealed new kinetic disturbances and a decline in LVEF of more than 20 %. Treatment was discontinued also due to progressive disease. The second patient developed a late onset decrease of LVEF from 65 to 35 % about 21 months after DRZ/DOX had been stopped. Three out of 27 patients who had received DOX before already met the criteria of functional AICM before initiating combination with DRZ. None of these patients later on developed CHF or further significant decrease in LVEF. In all patients, treatment could be safely carried out until the disease progressed. For example, one patient who met criteria of functional AICM with a decrease of LVEF from 77 to 56 % during previous anthracycline therapy, received 21 further cycles of DOX/DRZ (up to 1860 mg/m^2^ DOX cumulative dose without any further decline in LVEF).

Hence, all 32 patients were able to continue the combination of DOX and DRZ, until progressive disease occurred. Median DOX cumulative dose was 750 mg/m^2^ [120–1860 mg/m^2^] (Fig. 1).

### Subgroup of patients with DRZ at rechallenge with DOX therapy

Eleven patients met the criteria of rechallenge and were given DRZ from beginning of retreatment with DOX. The median DOX dose before retreatment was 450 mg/m^2^ [240–850 mg/m^2^]. Patients received a median of 5 cycles [1–21 cycles]. The median PFS during the first DOX containing regimen was 13 months [3–25 months]. At rechallenge, the median PFS with DRZ was 7 months [0–22 months; 95 % CI 3,7–10,2 months]. The clinical benefit rate was 91 % with 10/11 patients having at least stable disease at first evaluation.

### Subgroup of patients with DRZ in patients with continuous DOX therapy

**Fig. 3:**
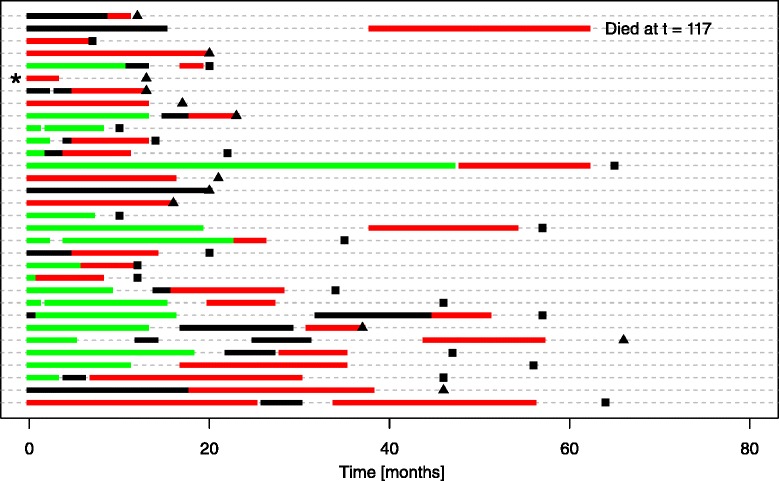
Illustration of administered chemotherapy regimens. Each row represents an individual patient. The length of the segments indicates the time from onset of the chemotherapy regimen to progression. Censoring and death events are marked by triangles and squares, respectively. Color coding: green = anthracycline, red = anthracycline + DRZ, black = other substance(s). Patients are sorted in ascending order of total administered anthracycline dose. The patient marked by an asterix received adjuvant chemotherapy before start of palliative treatment, which is not represented in the plot

Eighteen of 27 patients met the criteria of continuous dosing received DRZ during an ongoing DOX therapy at a median cumulative dose of 450 mg/m^2^ [260–585 mg/m^2^]. The reason of adding DRZ was at the discretion of the treating physician. There were no general recommendations about when to initiate prophylactic DRZ. Some of the patients had DOX in a stop-and-go fashion as an individual continuation therapy or were put on a drug holiday and DOX was reintroduced upon progressive disease or the onset of new symptoms. Patients then continued DRZ in combination for a median of 3,5 cycles [1–10 cycles]. The median PFS was 13 months [3–25 months; 95 % CI 8,8–17,2 months] from beginning of chemotherapy and 9 months from addition of DRZ [2– 21 months; 95 % CI 2,1–15,9 months]. The overall response was stable disease (SD) in 11/18 patients and partial response (PR) in 7/18 patients. At time of the evaluation, remission was ongoing for three patients (16+, 18+, 20+ months) (Fig. 3).

### Efficacy data for the whole cohort

The 32 patients of this retrospective analysis were treated with 58 anthracycline containing chemotherapy regimens altogether. The median PFS was 11 months [range: 0–47 months, 95 % CI: 7,6–14,3 months].

**Fig. 4:**
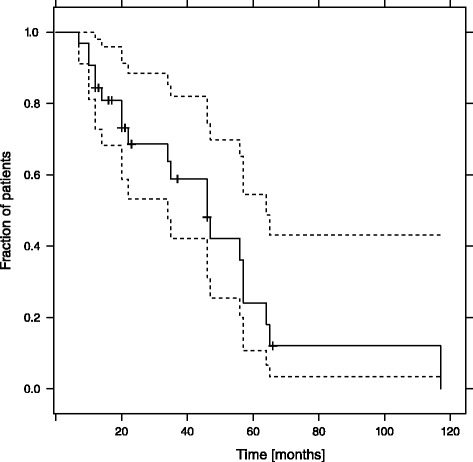
Estimated overall survival in all patients from the beginning of palliative chemotherapy. The dotted lines represent point-wise 95 % confidence intervals for the survival curve

**Fig. 5:**
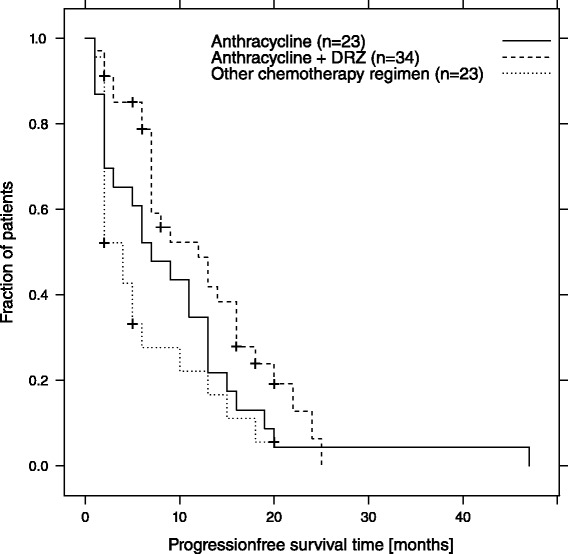
Estimated PFS-time in dependence of applied chemotherapy Anthracycline = Anthracycline containing regimen

The median OS from diagnosis of sarcoma was 57 months [range: 11–216 months; 95 % CI: 37,7–76,3 months]. The median OS from diagnosis of metastases was 47 months [range: 8–132 months; 95 % CI: 29,3–64,7 months]. The median OS from start of first anthracycline containing chemotherapy was 46 months [range: 3–83 months; 95 % CI: 30,3–61,7 months] (Fig. 4). The median PFS for all patients receiving DOX based therapy was 7 months (95 % CI 3–13 months), 12 months (95 % CI 7–16 months) for DOX/DRZ regimes and 4 months (95 % CI 2–13 months) for other regimens (Fig. 5). Nevertheless, these differences in PFS fail to reach statistical significance.

## Discussion

In this retrospective study, we evaluated the safety and outcome of patients with soft tissue sarcoma undergoing cardioprotective therapy with DRZ. This report demonstrates that high cumulative dosage of anthracyclines in combination with DRZ starting can be safely administered in sarcoma patients. Twenty-seven patients received DRZ from a median DOX cumulative dose of 450 mg/m^2^ onward. In all patients, DRZ/DOX containing therapy could be carried out until progression of the disease. Interestingly, 3 patients with AICM after prior DOX were able to continue DOX in combination with DRZ without any further deterioration of cardiac function. One patient experienced late-onset AICM. Altogether, the median cumulative anthracycline dose was 750 mg/m^2^ [330–1860 mg/m^2^].

Second, up to now there are no published data on the efficacy of reexposition of sarcoma patients with anthracyclines. In our series, patients rechallenging received a median of 5 further cycles with a median PFS of 7 months. So the clinical benefit rate was 91 % in patients that had achieved at least stable disease during prior treatment with DOX.

It is clearly not a clinical standard to exceed administration of DOX beyond 6 cycles. Even though DOX is still the most valuable cytotoxic agent in palliative treatment of sarcoma patients, there have never been systematic approaches to extend therapy. The reason not to extend DOX therapy is probably mainly explained by the known risk of cumulative cardiac toxicity. Nevertheless, for other agents, good safety has been demonstrated and clinically promising results could be achieved when the application was not confined to a definite number of cycles [31]. Therefore, in clinical practice, it seems feasible and promising to continue therapy and not only prolong time to progression in patients with no or little adverse effects. Taking into account that from the time of adding DRZ in all DOX pretreated patients PFS was 9 months, this seems to be an interesting option in patients who may benefit from anthracycline rechallenge. Of note, DOX was never used as a single agent in this series.

Third, the OS from the first commencing anthracycline containing therapy in our patients was 46 months and 17 months, from first introducing DRZ. Overall, our data support the hypothesis that extension of DOX in patients with advanced or metastatic soft tissue sarcoma will have a substantial clinical benefit by using DRZ in combination with an anthracycline-containing regimen. Although the criteria for initiation of DRZ in this series were not clearly defined, the OS of 17 months in a heavily pretreated subset of patients remains remarkable.

This study also has several limitations. First of all, the data were collected retrospectively and were also taken from only four institutions. Second, there might be a selection bias, even though the charts of all patients of the institutions were included in this series. Third, the time and the reason of introduction of DRZ in an ongoing regimen or upon rechallenge were not clearly defined. Therefore, the cumulative dose of DOX varies between 240 and 850 mg/m^2^ when the coadministration began, and almost all patients showed no functional or clinical signs of AICM at that time. As mentioned earlier, current guidelines recommend limiting DOX use to a cumulative dose of 450 mg/m^2^. Fourth, nowadays more therapeutic options beyond DOX, ifosfamide, dacarbazine, gemcitabine and docetaxel, are available, which offer a broader spectrum of agents to the physician.

## Conclusions

In conclusion, in our series adding DRZ to a DOX-based therapy can prevent AICM. Patients were able to receive a median of 750 mg/m^2^ DOX. Therapy could be safely carried out until disease progression. In all patients with benefit to the treatment, there was no need to discontinue DOX due to cardiac toxicity. The PFS of 9 months and corresponding OS of 17 months from the beginning of the coadministration of DRZ seem quite promising in this heavily pretreated group of patients. Prospective studies to confirm our findings are warranted.

## Abbreviations

AICM, anthracycline-induced cardiotoxicity; ASCO, American Society of Clinical Oncology; BC, breast cancer; CD, cumulative dose; CHF, congestive heart failure; CI, confidence interval; CIN, cardiac injury; DOX, doxorubicin; DRZ, dexrazoxane; LVEF, left ventricular ejection fraction; OS, overall survival; PFS, progression free survival; RC, rechallenge; RECIST 1.1, response evaluation criteria in solid tumors; TTE, transthoracal echocardiography
